# Antipsychotic-Associated Hypothermia With Bradycardia and QT Prolongation in an Older Adult

**DOI:** 10.7759/cureus.114939

**Published:** 2026-08-21

**Authors:** Iara Ferreira, Gonçalo Carneiro, Gonçalo Sarmento, Márcia Cravo

**Affiliations:** 1 Internal Medicine, Unidade Local de Saúde de Entre Douro e Vouga, Santa Maria da Feira, PRT

**Keywords:** adverse drug reaction, antipsychotics, bradycardia, hypothermia, qt interval prolongation

## Abstract

Second-generation antipsychotics may cause rare but potentially life-threatening disturbances in thermoregulation and cardiac conduction. We report a 92-year-old man with severe frailty, cognitive impairment, pre-existing sinus bradycardia, and right bundle branch block who presented on three occasions with recurrent hypothermia, progressive bradycardia, and altered mental status. The initial episode was attributed to an *Enterobacter cloacae* complex urinary tract infection. However, symptoms recurred despite negative repeat urine cultures, culminating in profound hypothermia, hypotension, a heart rate of 26 beats/min, and marked QT interval prolongation. No persistent infectious, endocrine, environmental, or acute neurologic cause was identified. Medication reconciliation subsequently confirmed a recent increase in quetiapine from 25 mg to 100 mg nightly, with concurrent risperidone administration. A suspected adverse reaction to second-generation antipsychotics was suspected, with advanced age, frailty, dehydration, and recent infection considered predisposing factors. Quetiapine and risperidone were discontinued, after which body temperature and the QT interval normalized within five days, heart rate returned to baseline, and mental status, renal function, leukocyte count, and platelet count recovered. This case highlights the importance of medication reconciliation and early recognition of antipsychotic-associated hypothermia in frail older adults.

## Introduction

Antipsychotic-associated hypothermia is an uncommon but potentially life-threatening adverse drug reaction that remains substantially less recognized than antipsychotic-associated hyperthermic syndromes. Hypothermia is defined as a core body temperature below 35°C and, as the temperature falls, may produce impaired consciousness, bradycardia, hypotension, hypoventilation, coagulopathy, and cardiac dysrhythmias. In older adults, these manifestations may be attributed to infection, endocrine disease, environmental exposure, or neurologic deterioration, potentially delaying recognition of a medication-related cause [1].

Evidence from published case reports and systematic reviews indicates that both first- and second-generation antipsychotics can impair thermoregulation at therapeutic doses [1]. The risk appears greatest during the first seven days after treatment initiation or dose escalation, although delayed episodes have also been described. Advanced age, sedative co-medication, hypothyroidism, and environmental cold exposure are among the reported predisposing factors [1]. The mechanism is likely multifactorial: interference with central serotonergic and dopaminergic pathways may disrupt hypothalamic temperature regulation, while alpha-adrenergic blockade, sedation, reduced physical activity, and impaired shivering may further limit heat conservation and heat production [1].

Causal attribution may be particularly challenging in frail older adults with polypharmacy and competing clinical explanations. Structured tools such as the Naranjo Adverse Drug Reaction Probability Scale provide a standardized framework for assessing the likelihood of a drug-related event by considering temporal relationships, dechallenge, rechallenge, and alternative causes [2].

Cardiovascular manifestations may reflect the combined effects of hypothermia and antipsychotic exposure. Progressive cooling itself may produce sinus bradycardia and conduction abnormalities, while antipsychotics can additionally affect autonomic tone and ventricular repolarization [1,3]. Severe hypothermia associated with bradycardia and cardiac arrest has been reported during risperidone therapy [4], and symptomatic bradycardia and hypotension have also been described with quetiapine in an older adult [5].

We report a frail 92-year-old man with pre-existing sinus bradycardia and right bundle branch block who developed recurrent hypothermia, profound bradycardia, altered mental status, and marked QT interval prolongation after a fourfold increase in the quetiapine dose and initiation of risperidone five days before the first episode. The close temporal relationship, recurrent clinical pattern, and subsequent improvement after withdrawal of both agents prompted formal causality assessment.

## Case presentation

A 92-year-old man with severe functional dependence, cognitive impairment, and known sinus bradycardia with right bundle branch block presented to the emergency department on three occasions with recurrent hypothermia, bradycardia, and altered mental status. He was receiving multiple chronic medications, including quetiapine and risperidone.

At the first presentation, his temperature was 33.4°C and heart rate was 54 beats/min. Urinalysis showed pyuria and positive nitrites, and urine culture grew *Enterobacter cloacae* complex. The clinical picture was initially attributed to a urinary tract infection. He improved after active external rewarming and antibiotic therapy and was discharged. Four days later, he returned with vomiting, worsening lethargy, recurrent hypothermia (32.6°C), and bradycardia (33 beats/min). Repeat urine culture was negative, and improvement after rewarming was again transient.

**Figure 1:**
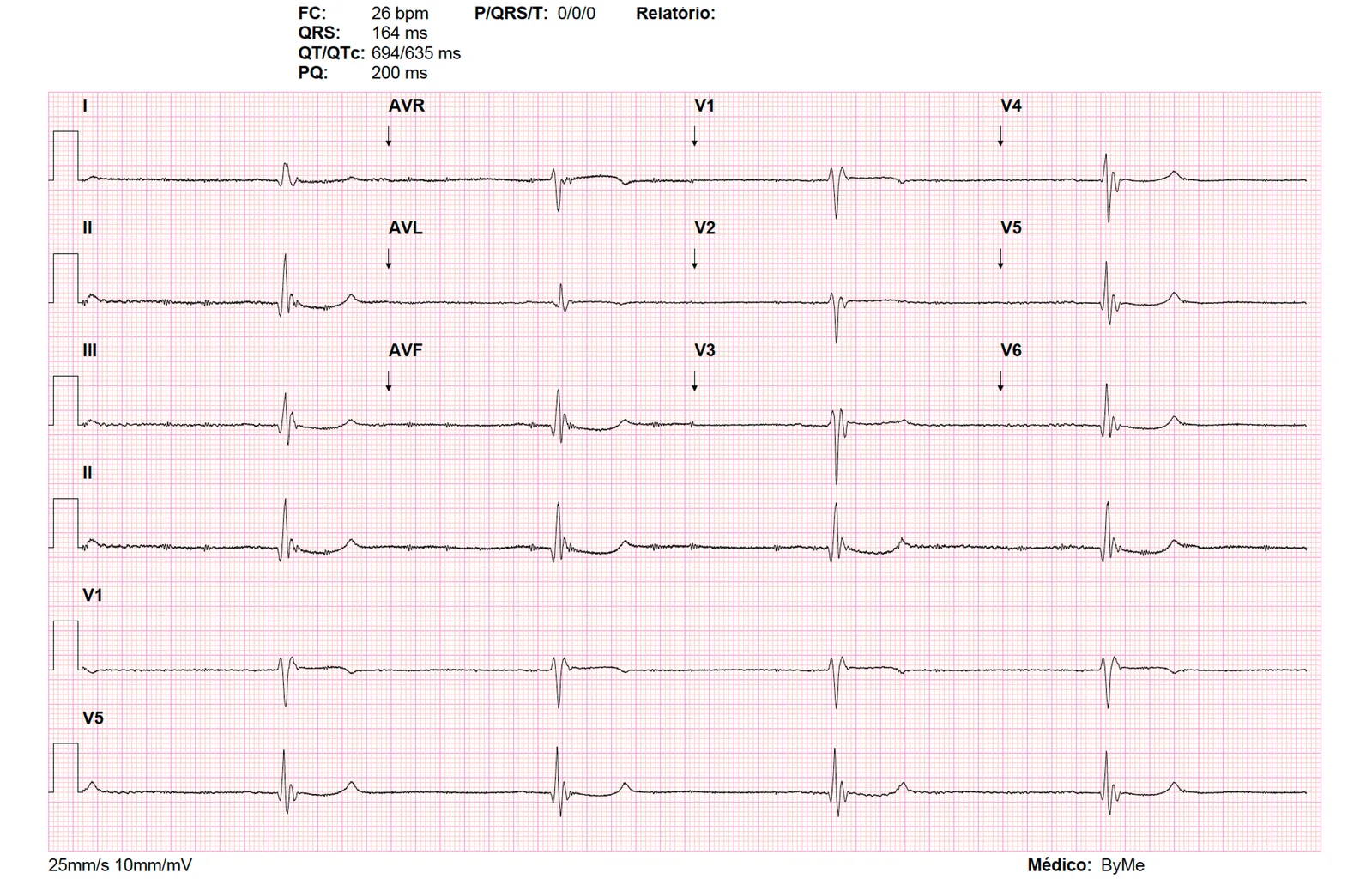
Electrocardiogram demonstrating profound sinus bradycardia and marked QT prolongation. Twelve-lead electrocardiogram obtained during the third emergency department presentation, showing profound sinus bradycardia at 26 beats/min, right bundle branch block with a QRS duration of 164 ms, and marked QT prolongation, with a measured QT interval of 694 ms and an automated corrected QT interval of 635 ms. Standard calibration was 25 mm/s and 10 mm/mV. QTc, corrected QT interval.

Five days later, he was readmitted with persistent lethargy, refusal of food and medication, and markedly reduced communication. He responded only to painful stimuli. His temperature was initially below the measurable range, blood pressure was 70/54 mmHg, and heart rate was 27 beats/min. Electrocardiography showed sinus bradycardia at 26 beats/min, the previously documented right bundle branch block, and new marked QT prolongation, with a measured QT interval of 694 ms and an automated corrected QT interval of 635 ms (Figure 1).

Laboratory testing showed leukopenia, thrombocytopenia, mild anemia, hypernatremia, Kidney Disease: Improving Global Outcomes (KDIGO) stage 1 acute kidney injury consistent with dehydration, and normal serum calcium (9.4 mg/dL) and magnesium (0.78 mmol/L) levels (Table 1).

**Table 1:** Laboratory findings at hospital admission. Reference ranges are those of the reporting laboratory. Capillary glucose was measured at triage. AST: aspartate aminotransferase; ALT: alanine aminotransferase; GGT: gamma-glutamyl transferase; LDH: lactate dehydrogenase; CK: creatine kinase; CRP: C-reactive protein; NR: not reported.

Parameter	Result	Unit	Laboratory reference range
Hemoglobin	11.1	g/dL	13.0–17.0
White blood cell count	3.4	× 10⁹/L	4.0–11.0
Lymphocytes	23.4	%	NR
Platelet count	100	× 10⁹/L	150–450
Sodium	146.0	mmol/L	136.0–145.0
Potassium	4.8	mmol/L	3.5–5.1
Corrected calcium	9.4	mg/dL	8.5–10.5
Magnesium	0.78	mmol/L	0.66–1.07
Capillary glucose	120	mg/dL	NR
Urea	118	mg/dL	18–55
Creatinine	1.6	mg/dL	0.7–1.3
Total bilirubin	0.58	mg/dL	0.20–1.20
AST	73	U/L	5–34
ALT	107	U/L	0–55
GGT	41	U/L	<55
Alkaline phosphatase	131	U/L	40–150
LDH	271	U/L	125–220
CK	179	U/L	NR
Myoglobin	295.1	ng/mL	NR
CRP	21.2	mg/L	<5.0
Urine leukocytes	125	/µL	<10
Urine nitrites	Negative	-	Negative
Urine culture	Negative	-	Negative

Lactate was normal, and repeat urine culture was negative. Active external rewarming, intravenous fluids, and continuous cardiac monitoring were initiated. Because of the recurrent and progressively severe episodes, alternative causes were investigated. Thyroid function, serum cortisol, and hemoglobin A1c were unremarkable (Table 2).

**Table 2:** Additional laboratory evaluation for secondary causes of hypothermia. Reference ranges are those of the reporting laboratory. TSH: thyroid-stimulating hormone; FT4: free thyroxine; HbA1c: glycated hemoglobin.

Parameter	Result	Unit	Laboratory reference range
TSH	4.09	µIU/mL	0.35–4.94
FT4	12.6	pmol/L	9.0–19.0
Morning serum cortisol	305.3	nmol/L	101.2–535.7
Serum glucose	78	mg/dL	83–110
HbA1c	5.6	%	<5.7
Corrected calcium	9.6	mg/dL	8.5–10.5
Magnesium	0.81	mmol/L	0.66–1.07

**Figure 2:**
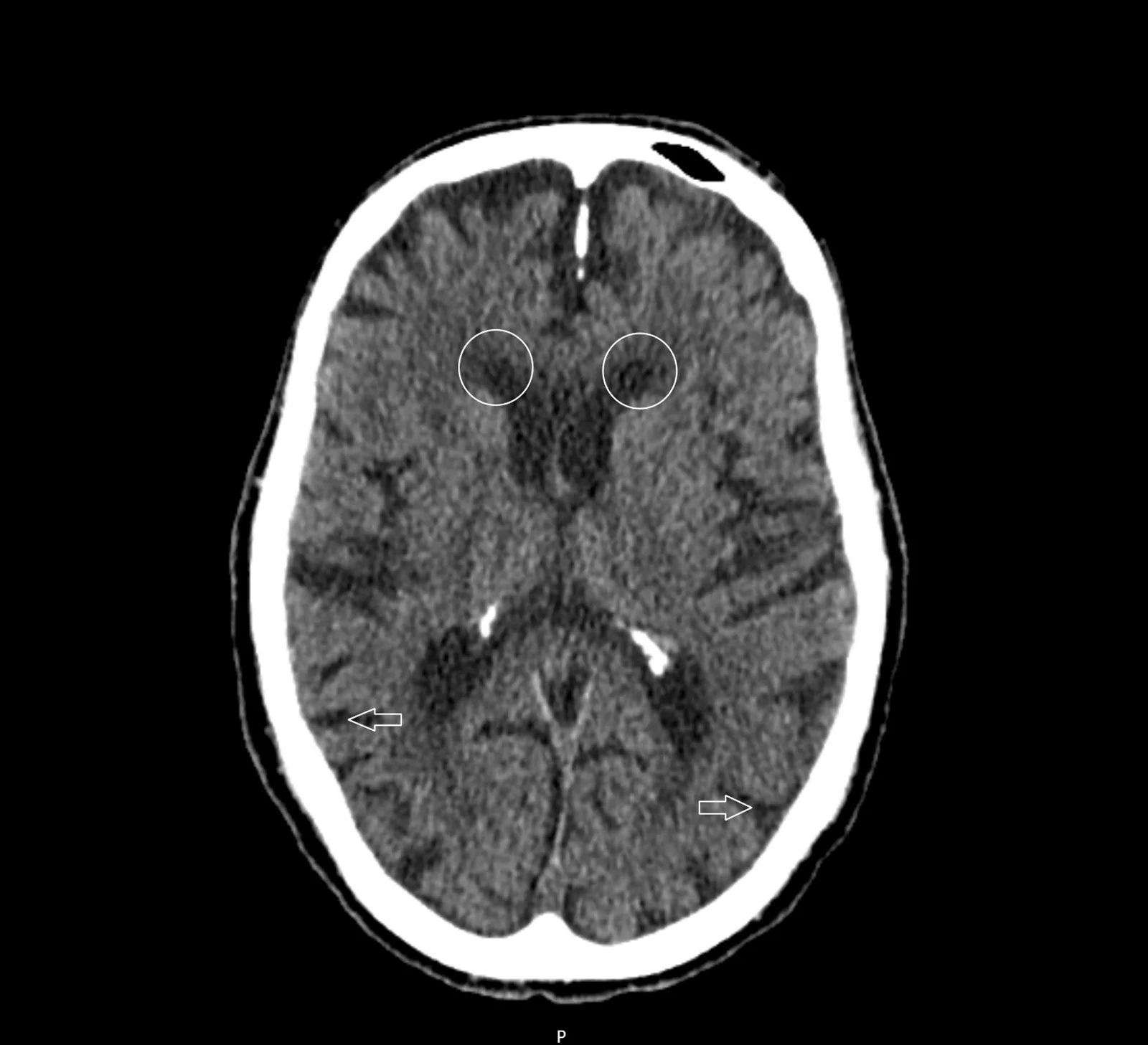
Non-contrast cranial computed tomography. Axial non-contrast cranial computed tomography showing bilateral periventricular hypodensities (open circles), compatible with chronic ischemic leukoencephalopathy, and prominent cortical sulci (arrows), consistent with cerebral atrophy. No acute intracranial abnormality is identified.

Cranial computed tomography showed chronic ischemic leukoencephalopathy and cerebral atrophy without acute abnormalities (Figure 2).

There was no environmental cold exposure, and no persistent infectious, endocrine, metabolic, or acute neurologic cause was identified. Medication reconciliation with the nursing home established that quetiapine had been increased from 25 mg to 100 mg nightly and risperidone 1 mg had been initiated five days before the first hypothermic episode. This close temporal relationship, together with the recurrent clinical pattern and newly documented QT prolongation, raised suspicion of an adverse reaction to second-generation antipsychotics.

Quetiapine and risperidone were discontinued. Over the following five days, QT prolongation resolved and the heart rate returned to his baseline range of 40-50 beats/min, while body temperature progressively improved. Mental status, renal function, serum sodium, leukopenia, and thrombocytopenia also improved. This clinical course was considered consistent with a positive dechallenge. A formal causality assessment using the Naranjo Adverse Drug Reaction Probability Scale yielded a score of 5 for quetiapine, corresponding to a probable adverse drug reaction, and a score of 4 for risperidone, corresponding to a possible adverse drug reaction [2]. Because both antipsychotics were administered concurrently and discontinued during the same clinical period, the individual contribution of each agent could not be definitively determined. Low-dose trazodone was later introduced for nocturnal agitation and insomnia. At discharge, the patient was normothermic, communicative, and hemodynamically stable, and further exposure to second-generation antipsychotics was discouraged.

## Discussion

This case illustrates a rare but potentially life-threatening suspected adverse reaction associated with the concurrent use of quetiapine and risperidone in a frail older adult. The initial urinary tract infection provided a plausible explanation for the first episode of hypothermia and altered mental status. However, the recurrence and increasing severity of hypothermia and bradycardia despite negative subsequent urine cultures, the absence of a persistent infectious, endocrine, environmental, or acute neurologic explanation, and the sustained recovery after antipsychotic withdrawal favored a medication-related contribution. The absence of recurrent marked hypothermia during a subsequent nosocomial infection provided additional support for this interpretation, although the infection may have acted as an initial precipitating factor.

Antipsychotic-associated hypothermia is uncommon, underrecognized, and potentially fatal. A systematic review identified 57 original cases occurring at nontoxic antipsychotic concentrations. In 58% of cases, hypothermia followed treatment initiation or dose escalation, and 69% of these episodes occurred within the first seven days. Advanced age, concomitant benzodiazepine use, subclinical hypothyroidism, and environmental cold exposure were the most frequently reported additional risk factors [1]. Risperidone was among the three agents most frequently represented in the review, although the reported frequencies cannot be interpreted as prevalence estimates or comparative risks. Severe hypothermia associated with bradycardia and cardiac arrest has also been described during risperidone therapy [1,4].

This patient had several factors that may have increased his susceptibility, including advanced age, severe frailty, limited mobility, cognitive impairment, polypharmacy, dehydration, recent infection, and concomitant zolpidem use. Medication reconciliation established a fourfold increase in quetiapine from 25 mg to 100 mg nightly and initiation of risperidone 1 mg five days before the first hypothermic episode, placing both medication changes within the highest-risk period described in the literature [1]. A structured causality assessment using the Naranjo algorithm yielded a score of 5 for quetiapine, corresponding to a probable adverse drug reaction, and a score of 4 for risperidone, corresponding to a possible adverse drug reaction [2]. The stronger individual signal for quetiapine was supported by the documented dose escalation; nevertheless, because both antipsychotics were used concurrently and discontinued during the same clinical period, their individual contributions cannot be definitively separated.

The mechanism of antipsychotic-associated hypothermia remains incompletely understood and is likely multifactorial. Antagonism of central serotonin 5-hydroxytryptamine-2 receptors may interfere with hypothalamic thermoregulation, whereas alpha-adrenergic receptor blockade may impair peripheral vasoconstriction and heat conservation. Sedation, reduced physical activity, and impaired shivering may further decrease heat production, particularly in older adults with limited physiological reserve [1]. In this patient, marked functional dependence and concomitant zolpidem use may have further impaired compensatory thermoregulatory responses.

The cardiac manifestations were also likely multifactorial. Severe hypothermia itself is associated with bradycardia and hypotension, whereas antipsychotics may independently affect heart rate and ventricular repolarization [1,3]. Quetiapine-associated symptomatic bradycardia and hypotension have been reported in an older adult, with sequential improvement following dose reduction and withdrawal [5]. Risperidone-associated hypothermia has similarly been described in conjunction with severe bradycardia and cardiac arrest [4]. In this case, pre-existing sinus bradycardia and right bundle branch block likely reduced the physiological tolerance of these effects. The profound bradycardia should therefore be interpreted as arising from the interaction between severe hypothermia, baseline conduction disease, and medication exposure rather than as an isolated antipsychotic effect.

The concurrent leukopenia and thrombocytopenia were considered potentially medication-related because both improved after discontinuation of quetiapine and risperidone. Reversible leukopenia and thrombocytopenia, with recurrence after quetiapine re-exposure, have previously been reported [6]. However, the available evidence is limited to case reports, and infection, systemic illness, hypothermia, and concurrent medications represent important confounding factors. A direct causal relationship therefore cannot be established. Persistent anemia was considered less likely to be medication-related because it did not resolve after antipsychotic withdrawal and was attributed to inflammation and folate deficiency.

The automated corrected QT interval of 635 ms should be interpreted cautiously because it was calculated during extreme bradycardia and in the presence of right bundle branch block. QRS widening increases the measured QT interval without necessarily reflecting an equivalent prolongation of ventricular repolarization, and automated correction methods may be unreliable in this setting [7]. Serum calcium and magnesium were within the reference range during the most severe episode, making clinically relevant hypocalcemia or hypomagnesemia unlikely contributors to the marked QT prolongation. Nevertheless, the newly documented measured QT interval of 694 ms, its clear difference from previous electrocardiograms, and subsequent resolution on serial recordings remained clinically relevant. Serial electrocardiographic changes were therefore considered more informative than the isolated automated corrected QT value.

Management of suspected antipsychotic-associated hypothermia includes gradual rewarming, correction of contributing metabolic and volume abnormalities, and close cardiorespiratory monitoring. Withdrawal or dose reduction of the suspected medication should be considered when there is a compelling temporal association or when severe clinical manifestations occur [1]. In this patient, discontinuation of both antipsychotics was followed by resolution of QT prolongation, recovery of the previous heart rate profile, progressive normalization of body temperature, and improvement in mental status and laboratory abnormalities. This clinical course was compatible with a positive dechallenge and supported a medication-related contribution. However, antipsychotic withdrawal occurred concurrently with active external rewarming, intravenous fluid replacement, antimicrobial treatment, and resolution of acute illness; therefore, the improvement cannot be attributed to drug withdrawal alone.

This case also emphasizes the importance of detailed medication reconciliation, particularly in institutionalized older adults whose complete medication history may not be immediately available. In susceptible patients, body temperature should be monitored after antipsychotic initiation or dose escalation, and electrocardiographic monitoring should be considered in the presence of cardiovascular disease, baseline bradycardia, conduction abnormalities, or other risk factors for QT interval prolongation [1,3]. Available evidence suggests monitoring body temperature for seven to 10 days after initiating an antipsychotic or increasing its dose in patients with relevant risk factors [1].

The main limitations of this report include the absence of antipsychotic serum concentrations, concurrent exposure to two suspected agents, simultaneous withdrawal of both drugs, and the absence of rechallenge. The exact administration frequency of risperidone and duration of zolpidem treatment were not fully available. In addition, the nursing home reported refusal of food and medication in the days preceding the third presentation, and the exact number of omitted antipsychotic doses could not be established. Rechallenge was not considered ethically appropriate given the severity of the presentation. Recent infection and dehydration remained plausible contributors to the hypothermia, while hypothermia itself may have contributed substantially to the profound bradycardia, QT changes, and hematologic abnormalities. The positive dechallenge was therefore interpreted as one component of the Naranjo assessment rather than as sufficient evidence by itself to establish causality [2]. Despite these limitations, the close temporal relationship, recurrent clinical pattern, objective electrocardiographic findings, and structured causality assessment support a probable contribution from quetiapine and a possible contribution from risperidone to the hypothermic syndrome.

## Conclusions

Antipsychotic-associated hypothermia is a rare but potentially life-threatening adverse reaction that should be considered in frail older adults presenting with recurrent hypothermia, bradycardia, altered mental status, or QT interval prolongation, particularly after treatment initiation or dose escalation. In this case, the close temporal relationship between antipsychotic exposure and symptom onset, recurrent episodes, objective electrocardiographic changes, and clinical improvement after drug withdrawal supported a medication-related contribution. Formal causality assessment classified the reaction as probable for quetiapine and possible for risperidone, although the individual contribution of each agent could not be definitively established. Prompt medication reconciliation, withdrawal of suspected agents, active rewarming, correction of reversible abnormalities, and cardiac monitoring are essential for the management of severe presentations. In vulnerable older adults, antipsychotics should be prescribed at the lowest effective dose, and their ongoing indication should be regularly reassessed.
